# APOE and gender influence cerebrospinal fluid lipids

**DOI:** 10.1002/alz.095144

**Published:** 2025-01-09

**Authors:** Angela J Hanson, Kristen M Farris, Jasroop K Miglani, Danijel Djukovic, Daniel Raftery

**Affiliations:** ^1^ University of Washington School of Medicine, Seattle, WA USA

## Abstract

**Background:**

High fat diets are a risk factor for Alzheimer Dementia (AD) but little is known about the effect of acute high fat feeding on brain lipid metabolism. Previous studies suggest that diet results may differ by APOE genotype and sex. Here we examined cerebrospinal fluid (CSF) lipidomic profiles after high and low fat feeding in a group of older adults to ascertain how APOE and sex influenced post‐prandial lipids.

**Method:**

As part of the Meal and Memory study (CT# NCT03070535), 78 healthy older adults (50% E4+, each E4 group 56% women) ingested high fat meal (HFM) and low fat meal (LFM) breakfasts 3‐5 weeks apart after overnight fast. Plasma triglycerides (TG) were measured at 7 time points, and lumbar puncture was performed 4 hours post‐meal. Lipidomics were performed on 250 ul of CSF on a Lipidyzer platform consisting of an AB Sciex 5500 MS/MS QTraps system equipped with a SelexION for differential mobility spectrometry (DMS). Multiple reaction monitoring was used to target 1,530 lipid species (across 20 lipid classes/subclasses) in positive and negative ionization modes with and without DMS, respectively. Lipids were analyzed using MetaboAnalyst 6.0 and SAS OnDemand (2021).

**Result:**

HFM expectedly increased plasma triglycerides (TG) (LFM peak TG 134±69 mg/dL, HFM peak TG 149±80, F 8.85, *p* = 0.004), results unaffected by E4 status or sex. No differences were found between the two meals for the 20 lipid classes or for individual lipid species in CSF. When examined by sex*APOE group, important baseline differences were noted in 10 of the 20 lipid classes. Furthermore, there were meal*group effects for phosphoserines (ANOVA F statistic 3.57, *p* = 0.024), with all groups except for E4+ men showing decreases in these lipids after HFM compared to LFM.

**Conclusion:**

Meals that produced predictable changes in plasma lipids did not change overall CSF lipids; however, changes were noted when analyzed by APOE*sex and further differed depending on whether the meal was high in fat. These findings suggest that diet or treatments that rely on brain lipid metabolism may need to be further stratified by APOE genotype and sex.

